# Editorial: Neurology and neuroimaging in exotic and non-domestic species

**DOI:** 10.3389/fvets.2022.1029791

**Published:** 2022-09-30

**Authors:** Silke Hecht, William B. Thomas, Michael Pees

**Affiliations:** ^1^Department of Small Animal Clinical Sciences, University of Tennessee College of Veterinary Medicine, Knoxville, TN, United States; ^2^Department of Small Mammal, Reptile and Avian Diseases, University of Veterinary Medicine Hannover, Hanover, Germany

**Keywords:** magnetic resonance imaging (MRI), relaxometry, rabbit, felid, bearded dragon, Kangaroo, central nervous system

Exotic animals including birds, reptiles and small mammals, have gained popularity as pets in recent years and are commonly presented for veterinary care to small animal practitioners and specialists. There is also increasing interest in conservation and optimized medical care of zoo and wildlife species. While many disorders of e.g., the respiratory, digestive and reproductive systems for certain exotic and non-domestic species are well described in the literature, information on neurologic conditions, their diagnosis, and treatment options is scarce. Diagnostic imaging of the central nervous system is an integral part in the work-up of neurologic conditions in human and veterinary patients. Certain information can be gained using survey radiography, contrast procedures (myelography), and computed tomography (CT). Magnetic resonance imaging (MRI) has revolutionized the field of neurology by not only providing excellent anatomic detail but also providing some functional information and allowing prognostication in certain diseases. The increasing availability of MRI scanners to veterinarians both in academic as well as specialty private practice settings has opened the door for neuroimaging research as well as optimized care of exotic veterinary patients.

This small but eclectic article collection includes a range of manuscripts from prospective hypothesis driven studies over retrospective case series to individual case reports, and covers a wide range of exotic and nondomestic animal species. Included publications not only provide important and clinically relevant information, they also provide a foundation for future related research projects.

An important contribution to the literature on advanced MRI techniques comes from Del Signore et al. who describe the use of a magnetic resonance-relaxometry (MRR) based technique for the identification of blood products in brain parenchyma in a rabbit model. The aim of this research study was to compare conventional low-field MR images with MRR in regards to lesion visibility and accuracy of lesion location compared to histopathology as the gold standard. A second aim was to test the performance of the developed classifier to differentiate healthy from abnormal tissue. The study shows that an advanced MRR protocol is promising for the detection of brain lesions even if using a low-field scanner, paving the way for possible future clinical use of this tool in veterinary practices.

A large-scale retrospective study by Hecht et al. provides an overview over the technique and findings in 50 nondomestic felids undergoing MRI of the brain and/or spinal cord. This is the largest case series on this topic to date. It not only includes descriptions of MRI abnormalities seen with common and well documented conditions such as Chiari-like malformation or intervertebral disc disease, but also with rare and previously unreported diseases such as various types of meningoencephalomyelitis, pituitary lesions, degenerative, congenital, metabolic, vascular, and traumatic conditions. This manuscript provides important information which will help to improve medical care of non-domestic felids with neurologic deficits in captivity, and may have future implications for work in conservation.

A research manuscript published by Foss et al. describes an MRI protocol which can be used for the evaluation of the brain in bearded dragons *in vivo*, and provides an atlas of normal anatomy. Imaging was performed using an injectable anesthetic protocol and allowed acquisition of a diagnostic quality MRI scan during an approximately 35-min timeframe. Images were acquired using a high-field 3T magnet, and the manuscript is beautifully illustrated with high quality labeled MR images of bearded dragon brains highlighting relevant anatomy. Bearded dragons are not only common in zoo collections, they are also increasingly popular companion lizards. In addition to providing a baseline for future research studies on neuroimaging in this species, this manuscript will therefore most certainly have practical implications for anybody performing and/or interpreting MRI studies in this species.

A very interesting case report by Huenerfauth et al. completes the article collection. Authors present imaging findings (MRI and CT) in a 10-year-old captive red Kangaroo presented with a chronic progressive pelvic limb lameness and reluctance to jump. Imaging revealed well-circumscribed mass lesions in the superficial erector spinae muscles, and histopathologic examination revealed metaplasia of muscle tissue to bone, consistent with myositis ossificans circumscripta. The authors report clinical improvement following a multimodal treatment approach including surgery, cage rest, weight reduction and medical management. This article not only provides further proof of the value of advanced imaging in the medical care of exotic animal species, it also includes important information on treatment options of this rather unusual condition.

With continued growth in awareness and increasing availability of advanced diagnostic imaging equipment in veterinary practice, it is likely that there will be an increasing demand for the diagnostic work-up and treatment of neurologic disorders in exotic animal species. This article collection provides a glimpse of the wide range of possible clinical applications in the field of exotic animal neurology, and opens the door for exciting new research paths.

## Author contributions

SH drafted the editorial. WT and MP reviewed the manuscript. All authors contributed to the article and approved the submitted version.

## Conflict of interest

The authors declare that the research was conducted in the absence of any commercial or financial relationships that could be construed as a potential conflict of interest.

## Publisher's note

All claims expressed in this article are solely those of the authors and do not necessarily represent those of their affiliated organizations, or those of the publisher, the editors and the reviewers. Any product that may be evaluated in this article, or claim that may be made by its manufacturer, is not guaranteed or endorsed by the publisher.

